# Methylprednisolone Induces Neuro-Protective Effects via the Inhibition of A1 Astrocyte Activation in Traumatic Spinal Cord Injury Mouse Models

**DOI:** 10.3389/fnins.2021.628917

**Published:** 2021-05-31

**Authors:** Hong-jun Zou, Shi-Wu Guo, Lin Zhu, Xu Xu, Jin-bo Liu

**Affiliations:** ^1^Department of Spinal Surgery, The Third Affiliated Hospital of Soochow University (The First People’s Hospital of Changzhou), Changzhou, China; ^2^Department of Cardiology, The Third Affiliated Hospital of Soochow University (The First People’s Hospital of Changzhou), Changzhou, China

**Keywords:** traumatic spinal cord injury, methylprednisolone, A1 astrocyte, AQP4, C3

## Abstract

Traumatic spinal cord injury (TSCI) leads to pathological changes such as inflammation, edema, and neuronal apoptosis. Methylprednisolone (MP) is a glucocorticoid that has a variety of beneficial effects, including decreasing inflammation and ischemic reaction, as well as inhibiting lipid peroxidation. However, the efficacy and mechanism of MP in TSCI therapy is yet to be deciphered. In the present study, MP significantly attenuated the apoptotic effects of H_2_O_2_ in neuronal cells. Western blot analysis demonstrated that the levels of apoptotic related proteins, Bax and cleaved caspase-3, were reduced while levels of anti-apoptotic Bcl-2 were increased. *In vivo* TUNEL assays further demonstrated that MP effectively protected neuronal cells from apoptosis after TSCI, and was consistent with *in vitro* studies. Furthermore, we demonstrated that MP could decrease expression levels of IBA1, Il-1α, TNFα, and C3 and suppress A1 neurotoxic reactive astrocyte activation in TSCI mouse models. Neurological function was evaluated using the Basso Mouse Scale (BMS) and Footprint Test. Results demonstrated that the neurological function of MP-treated injured mice was significantly increased. In conclusion, our study demonstrated that MP could attenuate astrocyte cell death, decrease microglia activation, suppress A1 astrocytes activation, and promote functional recovery after acute TSCI in mouse models.

## Introduction

Approximately half a million individuals worldwide are affected by traumatic spinal cord injury (TSCI) each year. The high prevalence is associated with significant personal and socio-economic impacts (Singh et al., 2014). After TSCI, tissues in the spinal cord induce self-destructive mechanisms termed secondary damage (Romanelli et al., 2019; Pelisch et al., 2020). The main mechanisms of secondary damages after TSCI are excitotoxicity, excessive free radical production, inflammation, and apoptosis (Diaz-Ruiz et al., 2009; Novgorodov et al., 2019). Studies that have focused on reducing neuroinflammation by promoting neuron survival and axon outgrowth have shown some satisfactory therapeutic efficacy (Sun et al., 2018; Lima et al., 2019). Methylprednisolone (MP) is a glucocorticoid drug that has been used in the clinical treatment of SCI due to its highly effective anti-inflammatory properties (Hall and Braughler, 1981). Studies have demonstrated that MP has neuro-protective properties against SCI by inhibiting microglia/macrophage accumulation, reducing calcium influx, increasing blood flow, and protecting glia from dysmetabolic insults (Tang et al., 2015; Keles et al., 2019; Samano and Nistri, 2019).

Astrocytes make up the majority of glial cells in the central nervous system (CNS). Astrocyte activation supports neuronal cells by secreting neurotrophic factors under different physiological conditions. However, the function of MP on astrocyte activation has been controversial. Astrocyte activation after nerve injury initiates the scarring process, which results in a long-lasting physical and chemical barrier to axonal regrowth during the chronic SCI phase (Wanner et al., 2008). MP has been shown to promote neurite outgrowth after excitotoxic insult through the glucocorticoid receptor-mediated downregulation of astrocyte reactivation and inhibition of CSPG expression (Liu et al., 2008). Astrocyte activation after TSCI has also been shown to be involved in the development of spinal cord edema, and MP may function in alleviating edema. The mechanism of astrocyte function is to decrease AQP4 expression and inhibit apoptosis (Lu et al., 2016). Recent studies have demonstrated the presence of two subtypes of astrocytes after CNS injury, i.e., A1 type astrocytes (A1s) and A2 type astrocytes (A2s) (Zamanian et al., 2012). A1s have lost normal glial cell function. They function to secrete neurotoxins to eliminate axonal injured neurons (Liddelow et al., 2017). A1s have been shown to migrate to the injured area after SCI (Wang et al., 2018, 2021) and participate in the neurotoxin process of nerve injury. Studies have shown that activated microglia could induce the transformation of naïve astrocytes into A1 astrocytes.

We hypothesized that MP could effectively suppress microglial activation and suppress astrocyte transformation to the A1 phenotype. To support our hypothesis, we analyzed the expression levels of IBA-1 (a specific marker of activated microglia) and complement C3 (a specific marker of A1 astrocytes), as well as the expression levels of TNFα (a pro-inflammatory cytokine) and IL-1α (a pro-inflammatory cytokine), which are essential for inducing A1 astrocytes. Our results demonstrated that MP could reduce astrocyte cell death, inhibit microglial activation, suppress A1s activation, and regulate axonal regeneration, resulting in the functional recovery of mice after TSCI.

## Materials and Methods

### Experimental Animals

BALB/c mice, 6–8 weeks old, were purchased from the Changzhou CAVENCE experimental center and maintained in SPF level conditions. Water and food were provided ad libitum. All experiment procedures followed the ethical guidelines for animal research developed by the science and technology department of China and were approved by the ethics committee for animal research, Third Affiliated Hospital of Soochow University.

### Astrocyte Primary Culture and Identification

Based on the Institutional Animal Care and Use Committee guidelines of Soochow University, primary astrocytes were isolated from BALB/c mice on postnatal days 1–3. Astrocytes were harvested as follows: Newborn BALB/c mice (within 72 h) were disinfected by soaking in 75% alcohol for several seconds. Afterward, the mice were decapitated with tissue scissors and the whole brain was excised using ophthalmic curved tweezers. The brain tissues were then placed in precooled serum-free medium Dulbecco’s Minimum Essential Medium (DMEM) and the meninges, blood vessels, and hippocampus were removed under a stereomicroscope to obtain complete cerebral cortex tissue. The tissues were then minced using a scalpel and then incubated and digested with type I collagenase and 0.25% pancreatin separately for 15 min, with gentle mixing every 5 min, and vortexing at 2000 r/min for 90 s. The suspension was then passed through a 70 μm nylon mesh, and 10% FBS complete medium was added to the cell suspension. Purification was performed by shaker. The purifiedcell was then cultured in a 35 mm plastic petri dish with growth media [DMEM containing 10% fetal bovine serum, penicillin (10 units/ml), streptomycin (10 mg/ml), and L-glutamine (29.2 mg/ml)]. The media was changed every 3 days.

Cell growth was observed under an inverted phase-contrast microscope and photographed. After 3–4 weeks of continuous culture, a portion of the cells was washed in PBS buffer three times, fixed in 4% polyformaldehyde for 30 min, washed in PBS buffer three times, and permeabilized in 0.25% TritonX-100 at room temperature for 15 min. Afterward, the cells were washed in PBS buffer three times and incubated with 3% BSA at room temperature for 2 h. Cells were then incubated in rabbit anti-GFAP polyclonal antibody (1: 100) or rabbit anti-S100β polyclonal antibody (1: 100) overnight at 4°C. The next day, the primary antibody was removed, and the cells were washed in PBS buffer three times. Cells were then incubated with secondary anti-sheep anti-rabbit FITC-IgG (1: 100) at 37°C for 30 min. Cells were stained with DAPI for 20 min and then sealed with glycerol film and observed and photographed using a fluorescence microscope.

### Astrocyte Apoptosis Assays

Astrocytes were incubated with or without 10 μg/ml of MP (Pfizer Inc., New York, NY, United States) after treatment with hydrogen peroxide (H_2_O_2_; 100 mM) for 24 h (Vieira et al., 2008). Cells were digested with trypsin without EDTA. Afterward, the incubation was terminated, and the cells were collected, centrifuged at 1,000 rpm at 4°C for 5 min, and the supernatant was discarded. The cells were washed twice with pre-cooled PBS, centrifuged at 1,000 rpm at 4°C for 5 min each time, and the supernatants were discarded. Cells were then resuspended and incubated in Annexin V-PE (Vazyme) and 7-AAD for 5 min at room temperature in the dark. The cells were then washed in PBS three times before being analyzed using a FACSVerse flow cytometer (BD Biosciences) running the FACSuite software. Data analysis was performed using the FlowJo software (Treestar).

### Western Blot Analysis

Western blot analysis was performed as previously described (Zou et al., 2015). Astrocytes were rinsed in cold PBS and then lysed in Radio-Immunoprecipitation Assay (RIPA) lysis buffer at 4°C for 10 min. Cell lysates were then centrifuged at 4°C, 12,000 r/min for 15 min, and protein concentration was determined in the supernatants using the BCA method. Equal amounts of proteins were separated using a 10% or 12% Sodium Dodecyl Sulphate–Polyacrylamide Gel Electrophoresis (SDS–PAGE). Separated proteins were then transferred to a Polyvinylidene Fluoride (PVDF) membrane and blocked with 5% non-fat milk powder for 1 h at room temperature. Membranes were incubated with the following primary antibodies: cleaved caspase-3 (1:1,000, rabbit IgG; Cell Signaling Technology, BSN, United States), B-cell lymphoma 2 (Bcl-2; 1:1000, rabbit IgG; Cell Signaling Technology, BSN, United States), Bcl-2–associated X protein (Bax; 1:1,000, rabbit IgG; Cell Signaling Technology, BSN, United States), C3 (1:2,000, rabbit IgG; Abcam, United Kingdom), or beta-Actin (β-actin; 1:1,000, mouse IgG; Cell Signaling Technology, BSN, United States). The membranes were then washed and incubated with secondary antibody (1:2,000; Thermo Pierce, MA, United States) for 120 min at RT. β-actin was used as the loading control (Thermo Pierce, MA, United States).

### Traumatic Spinal Cord Injury Mouse Model and Animal Care

**FIGURE 1:**
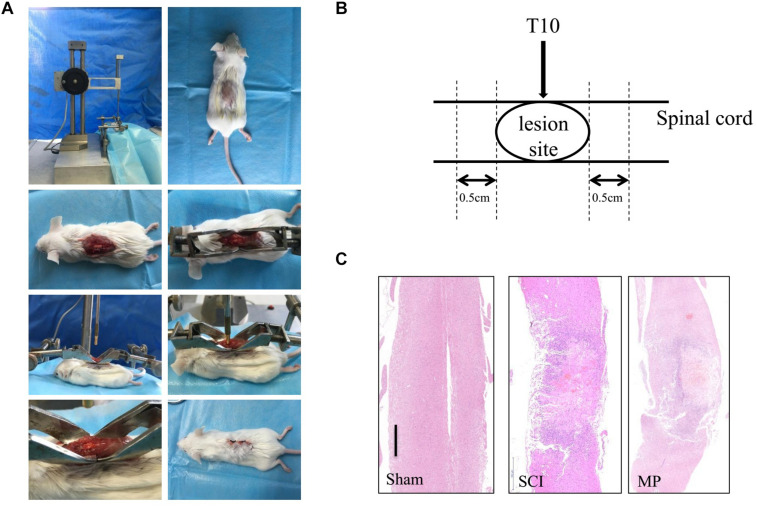
Mouse traumatic spinal cord injury. **(A)** Laminectomy was performed at the T9-10 vertebrate, and the exposed spinal cord (T10) was impacted with a 5-gram rod dropped 12.5 mm by NYU Impactor. Following removal of the rod, the muscles and skin were sutured in layers. **(B)** The operation schematic diagram. **(C)** Gross morphology and representative hematoxylin-eosin staining sagittal sections. Scale bar: 500 μm. *N* = 10 for each experiment. Sham group, which underwent laminectomy without TSCI; SCI group, which underwent laminectomy followed by TSCI and received saline i.v. immediately after injury; MP group, which underwent laminectomy followed by TSCI and received a 30 mg/kg dose of MP (Pfizer Inc., United States) i.v. immediately after TSCI via the tail vein injection.

BALB/c mice were anesthetized (i.p) using Nembutal (pentobarbiturate sodium, 0.25% W/V). The surgical procedure was performed at 38°C. Laminectomy was performed on the T9–10 vertebrate and the T10 spinal cord was injured using a 5-gram rod dropped from 12.5 mm using the NYU Impactor (New York University, New York, NY, United States). The wound was closed using 3-0 silk thread (Figure 1) (Young, 2002). After the procedure, mice were placed in warm cages with food and water. Post-injury bladder management was performed twice a day until bladder reflex recovery. Mice in the MP group were administered MP via the tail vein (30 mg/kg) and an equal volume of 0.09% saline was administered to the control group.

### Experimental Groups

Thirty mice were used in this study. Mice were randomly allocated into three groups (*n* = 10): The sham group underwent laminectomy without TSCI; the SCI group underwent laminectomy followed by TSCI and received saline i.v. immediately after injury; and the MP group underwent laminectomy followed by TSCI and was administered 30 mg/kg dose of MP (Pfizer Inc., United States) i.v. immediately after TSCI via the tail vein. MP was dissolved in phenylcarbinol and further diluted in physiological saline. On days 1, 3, 7, 14, 21, and 28 following trauma, five mice from each group were used for the analysis of functional recovery, i.e., determined using Basso Mouse Scale (BMS) scores and Footprint Tests. At 3 days and 28 days post-surgery, tissues from five mice per group were used for immunohistochemical and immunofluorescence staining.

### Immunohistochemical and Immunofluorescence

The immunohistochemical staining procedure was followed as previously described (Ge et al., 2013). Immunofluorescence staining was used to measure expression levels of NF-200 and GAP43 in the injured spinal cord at 3 days and 28 days post-injury. In addition, expression levels of IBA1, AQP4, IL-1α, and TNFα and C3 positive cells in the injured spinal cord were determined at 3 days post-injury. Mice were perfused transcardially with saline, then with 4% paraformaldehyde in phosphate-buffered saline (0.1 M PBS, pH 7.4). One cm long segments of the cord at the injury area were fixed for further study. The fixed cords were coated with paraffin and then axially sliced. Slices were then deparaffinized and treated with 3% H_2_O_2_ for 15 min to block endogenous peroxidase. After blocking with serum for half an hour, the slices were incubated with the following primary antibodies overnight at 4°C: rabbit polyclonal anti-AQP4 antibody (Cell Signaling Technology, Inc.), anti-NF-200 antibody (Cell Signaling Technology, Inc.), anti-TNFα antibody (Servicebio, Inc.), anti-IL-1α antibody (Proteintech, Inc.), anti-GAP43 antibody (Cell Signaling Technology, Inc.), and anti-C3 antibody (Abcam, Inc.). The slices were then washed with PBS three times and incubated with the appropriate secondary antibody (Boster, China) at 37°C for 15 min. Cell nuclei were stained with DAPI (Life Technologies). Stained cells were observed using an inverted fluorescence microscope (Leica, Germany).

### *In vivo* Apoptotic Assays (TUNEL Staining)

Terminal deoxynucleotidyl transferase dUTP nick end labeling (TUNEL) staining was performed to measure neuronal cell apoptosis in the TSCI area based on the manufacturer’s instructions. Briefly, cells were stained with TUNEL reaction mixture (Servicebio, Inc.) at 37°C for 30 min in the dark and then the nuclei were counterstained with DAPI for 5 min. The proportion of TUNEL-positive neurons was counted from randomly selected fields of view under a fluorescence microscope (Leica, Germany).

### Fluoro-Jade B Staining

Spinal cord sections were stained with Fluoro-Jade B to observe neuronal degeneration after TSCI (Anderson et al., 2003; Ahn et al., 2019). The spinal cord was immersed in 1% sodium hydroxide, transferred to 0.06% potassium permanganate, placed in 0.0004% F-J B (Servicebio, Inc.), and then dried using a slide warmer (approximately 50°C) for at least 5 min.

### Quantitative and Qualitative Analyses

For quantitative analyses, immunohistochemical counts generated by two technicians were averaged to obtain the final counts per section. The percentage of positive cells was rated as follows: 2 points, 11–50% positive cells; 3 points, 51–80% positive cells; and 4 points, > 81% positive cells. The staining intensity was rated as follows: 1 point, weak intensity; 2 points, moderate intensity; and 3 points, strong intensity. Points for expression and percentage of positive cells were added, and tissue samples were attributed to four groups based on their overall scores: negative, ≤10% of cells stained positive, regardless of intensity; weak expression, 3 points; moderate expression, 4–5 points; and strong expression, 6–7 points (Goh et al., 2008).

The number of positive cells or the mean optical density (mean optical density = integrated optical density [IOD]/area) as measured using Image J (version 1.48) was used for quantitative analyses for immunofluorescence. The number of IBA1 positive cells or the normalized mean optical density of IL-1α, TNFα, and C3 in the central canal of the spinal cord adjacent to the injury site was calculated.

For qualitative analyses of AQP4 polarity distribution, immunofluorescence staining for the water channels AQP4 and GFAP were used as described previously (Liddelow et al., 2017). Robust AQP4 protein localization to astrocytic endfeet on blood vessels was termed polarity distribution, while the loss of AQP4 immunoreactivity on astrocytic endfeet on blood vessels with increased staining in other regions of the astrocyte was termed loss of polarity.

### Functional Evaluation

The BMS scale was used as described in previous publications (Basso et al., 2006). Using this scale, specific components of functional behavior, such as ankle movements, stepping pattern, coordination, paw placement, trunk instability, and tail position, were measured and quantitated, with a minimum score of 0 (no movement) to a maximum score of 9 (normal locomotion). A 100 × 100 cm transparent plexiglass box was used to determine BMS scores. Two blinded observers were used to record movements for 5 min (Song et al., 2018).

For the Footprint Test, a 50 cm runway was used to evaluate mouse walking. The hind paws were marked with red ink during the footprint test. The stride length on each side and stride width between the two sides of the prints were recorded.

### Statistical Analyses

Comparison between the SCI and MP groups was performed using Student’s unpaired t-test or one-way ANOVA. *p*-values < 0.05 were considered statistically significant. Values in graphs are shown as mean ± standard error of the mean (SEM).

## Results

### Methylprednisolone Reduces *in vitro* Astrocyte Cell Death

**FIGURE 2:**
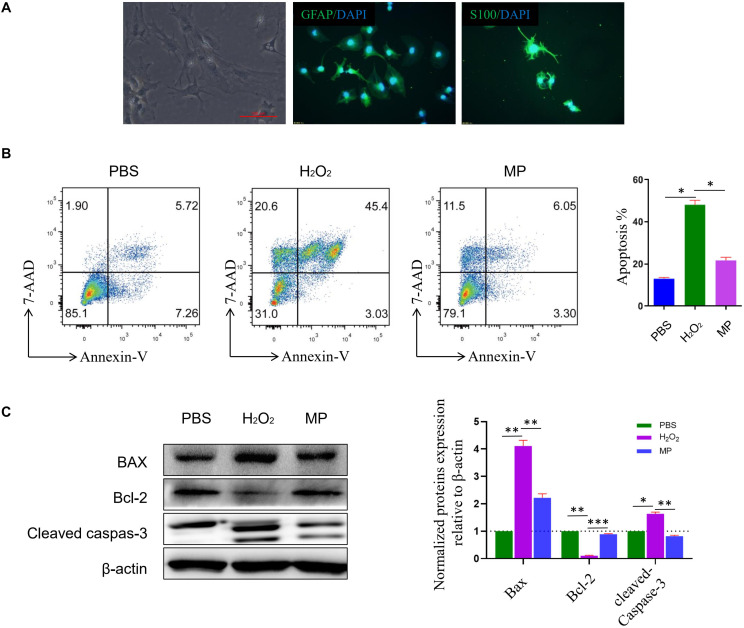
MP alleviates H_2_O_2_-induced astrocyte apoptosis *in vitro*. **(A)** Primary culture of astrocytes. GFAP and S100ß are used as astrocyte markers. DAPI is used for counterstaining the nuclei of living cells. The purity of the culture was calculated as the percentage of GFAP positive cells in the total number of cells (based on DAPI staining). Astrocytes account for about 95% of the total culture. **(B)** Astrocytes apoptosis induced by H_2_O_2_ together with or without MP (10 μg/ml) administration was assessed through Annexin V/7-AAD double staining with flow cytometric analysis. Quantitative results of apoptotic astrocytes with and without treatment by MP. ^*^P < 0.05. **(C)** Western blot analysis of apoptosis-related proteins (Bax, Bcl-2-associated X protein; Bcl-2, Bcell lymphoma 2; cleaved caspas-3) in astrocytes incubated with MP treatment after H_2_O_2_-induced oxidative damage. Semiquantification of relative expression levels of apoptosis-related proteins in primary astrocytes normalized to ß-actin. Scale bar: 100 μm. One-way ANOVA. Values are all expressed as mean ± SD. ^*^P < 0.05. ^**^P < 0.01. ^***^P < 0.001.

Primary cultured astrocytes generally adhere to the cell culture dish in approximately 2–3 days, and gradually form protrusions from the cell body. Cellular impurities can be removed by shaking uniformly and forcefully during cell culture media change. After 7 days of cell culture, the number of astrocytes increased significantly and formed protrusions that increased in length. Multiple protrusions made contact to form a neural network. Subsequently, mature astrocytes were formed that were large, irregular in shape, and rich in cytoplasm. Primary cultured astrocytes were identified using astrocyte markers GFAP and S100β. DAPI was used to counterstain the nuclei. The purity of the culture was calculated as the percentage of GFAP positive cells in the total number of cells (based on DAPI staining). Astrocytes accounted for about 95% of the total culture (Figure 2A). Previous studies have demonstrated that H_2_O_2_ could induce astrocyte cell death, while our study demonstrated that 10 μg/ml of MP could reduce astrocyte cell death after H_2_O_2_ exposure. Cells were stained with Annexin V-PE/7-AAD and run on a flow cytometer to assess early and late apoptosis rates. As shown in Figure 2B, exposure to H_2_O_2_ dramatically increased apoptosis compared to the control group, whereas administration of MP significantly attenuated the apoptotic effect of H_2_O_2_ in neuronal cells. Western blotting demonstrated that the level of apoptotic related proteins, Bax, and cleaved caspase-3 were reduced, while Bcl-2 was increased upon co-treatment with MP after 24 h (Figure 2C).

### Methylprednisolone Promotes Functional Recovery in TSCI Mouse Models

**FIGURE 3:**
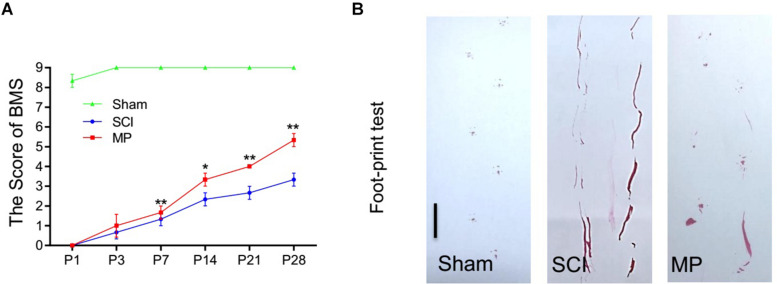
MP promoted functional behavioral recovery. **(A)** BMS grading scale was used to functionally grade the mice in both groups up to 28 days post-injury. **(B)** Footprint data in false-color mode. The footprint distance could not be measured consistently because the animals dragged their legs. Scale bar: 2.5 cm. MP group consistently exhibited significantly higher BMS scores compared with the SCI groups. Student’s unpaired *t*-test. Values are all expressed as mean ± SD. ^*^P < 0.05. ^**^P < 0.01.

Methylprednisolone attenuates neuronal cell death and promotes functional recovery after TSCI. The locomotor function of the hind limbs in the sham group recovered to a score of 21 at 3 days post-injury. In comparison, the BMS scores for the other groups were <2. Functional recovery was observed from days 7 to 28 post-injury. MP-treated mice showed significantly greater improvement in neurological function compared to mice administered saline (Figure 3A). Furthermore, mice treated with MP had significantly higher scores compared to mice administered saline from day 14 to the end of the study. A footprint test was performed 4 weeks after TSCI. The footprint distance could not be measured consistently because some of the mice dragged their legs. From the footprint images, we found that the motor function of the hind limbs in the MP group was significantly improved compared to the SCI group (Figure 3B).

### Methylprednisolone Suppresses Microglial Activation and Attenuates *in vivo* Neuronal Cell Death After TSCI

**FIGURE 4:**
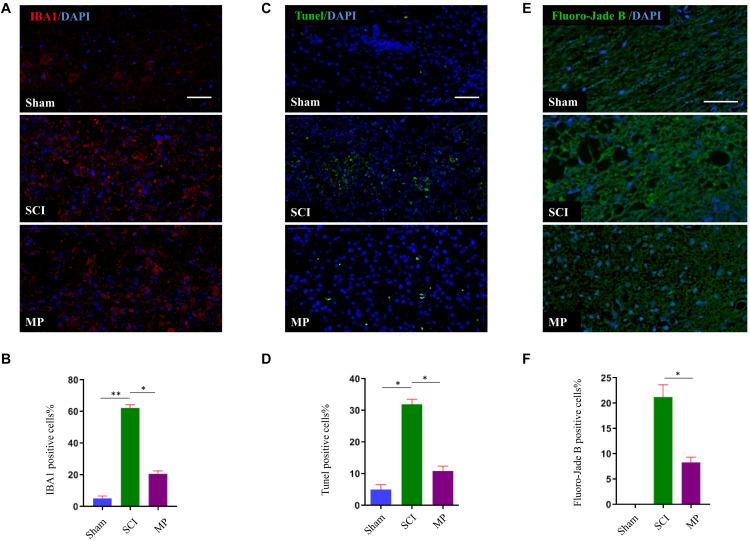
MP suppresses the activation of microglia and attenuates neuronal cell death in spinal cord injury *in vivo*. **(A)** Representative immunohistochemical staining images of IBA1 (red) in injured spinal cord tissue of the Sham, TSCI, and MP groups at day 1 post-injury. Nuclei of all cells were stained with DAPI (blue). **(B)** Analysis of the number of IBA1^+^ microglia in the traumatic lesion area. **(C)** Representative images of TUNEL-positive apoptotic cells (green) in sagittal spinal cord sections at day 1 post-injury. Nuclei of all cells were stained with DAPI (blue). **(D)** Comparison of the number of TUNEL-positive cells. **(E)** Representative images of Fluoro-Jade B cells (green) in sagittal spinal cord sections at day 1 post-injury. Nuclei of all cells were stained with DAPI (blue). **(F)** Comparison of the number of Fluoro-Jade B-positive cells. Scale bar: 50 μm. Student’s unpaired *t*-test. Values are all expressed as mean ± SD. ^*^P < 0.05. ^**^P < 0.01.

To evaluate the effects of MP on microglial activation after TSCI, we performed immunofluorescence staining for IBA-1 (a specific marker of activated microglia) adjacent to the injury lesion site. On day 1 after TSCI, the cell body of microglia was enlarged, the number of processes, and the number of IBA-1 positive cells increased (Figures 4A,B). The SCI group was administered a 30 mg/kg dose of MP i.v. immediately after TCI via tail vein injection. A significant reduction in IBA-1 expression was observed in the SCI group. In addition, the number of IBA-1 positive cells was lower compared to the SCI group. Neuronal cell apoptosis in the TSCI area was measured using TUNEL assays. On the first day after injury, the number of TUNEL-positive (apoptotic) cells in the MP group was significantly lower compared to the SCI group (Figures 4C,D). *In vivo* TUNEL assays further demonstrated that MP could effectively protect neuronal cells from apoptosis after TSCI, which was consistent with our *in vitro* experiments. Fluoro-Jade B staining was used to detect the presence of degenerative neurons. Our results showed that the number of Fluoro-Jade B cells in the MP group was significantly lower compared to the SCI group, while no Fluoro-Jade B positive cells were observed in the sham group (Figures 4E,F).

### Methylprednisolone Promotes *in vivo* Neuron Axonal Outgrowth

**FIGURE 5:**
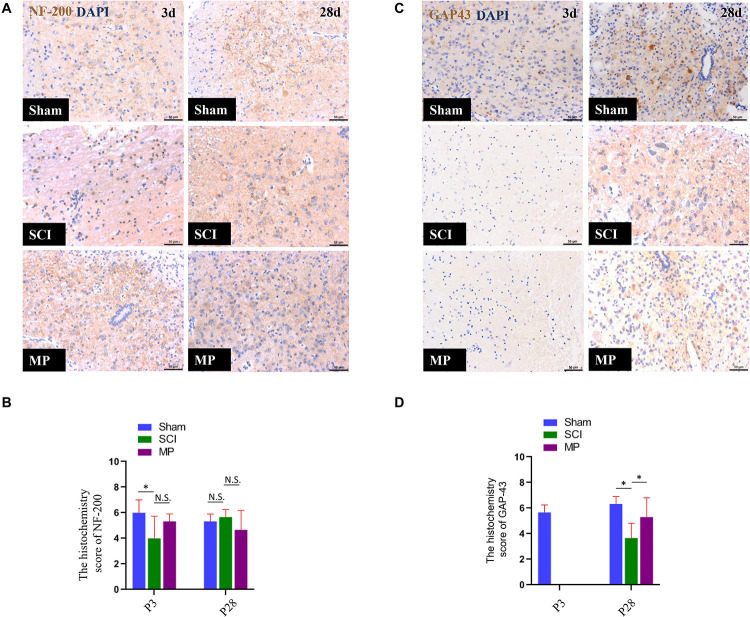
MP promotes neuron axonal outgrowth *in vivo*. **(A,C)** GAP-43 and NF-200 expression in the Sham group, SCI group, and MP group at day 3 and day 28. The number of GAP-43 positive cells in MP group was upregulated compared with that of TSCI group at 28 days after injury. However, the expression of NF-200 did not show significantly changes same as GAP-43. **(B,D)** The quantification of IHC results. Scale bar: 50 μm. Student’s unpaired *t*-test. Values are all expressed as mean ± SD. ^*^P < 0.05. N.S.: none significance.

Immunohistochemistry was used to investigate the effect on neurons and axons after MP treatment at 3 days and 28 days after TSCI (Figure 5). Previous studies have demonstrated a strong association between neurite outgrowth and GAP-43 expression levels (Zou et al., 2015). Hence, we measured GAP-43 levels to evaluate axons. Our results showed that the axons retreated from the edges of the lesion site over time after TSCI. The MP group showed higher GAP-43 expression levels compared to the SCI group (Figures 5C,D). To investigate the pathological changes and the effect of MP on neurons after TSCI, we measured NF-200 expression levels at the edges of the lesion site at 3 days and 28 days after TSCI (Figures 5A,B). However, NF-200 expression levels were not significantly different between the two groups.

### Methylprednisolone Suppresses A1 Astrocyte Activation

**FIGURE 6:**
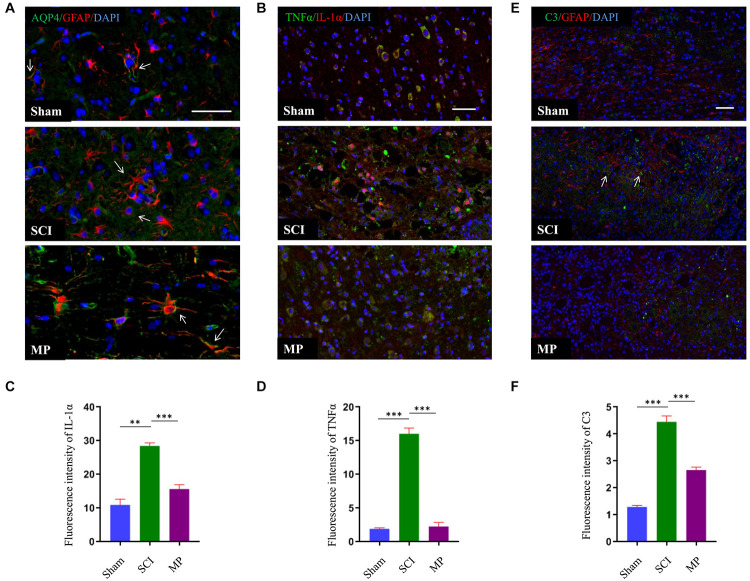
MP suppressed activation of A1 neurotoxic reactive astrocytes. **(A)** A1 astrocytes were identified by distribution of AQP4. The AQP4 (green) retained polarization localization in astrocytes GFAP (red) after MP injected compared to TSCI mice at day 3 (marked with arrow). **(B)** Representative immunohistochemical staining images of IL-1α (red) and TNFα (green) in the injured spinal cord tissues of TSCI and MP groups at day 3. **(C,D)** Quantitative results of the mean immunofluorescence density for IL-1α and TNFα with and without treatment by MP. **(E)** Representative immunohistochemical staining images of C3 (green)and GFAP (red) in the injured spinal cord tissues of Sham, TSCI, and MP groups at day 3 (marked with arrow). **(F)** Quantitative results of the mean immunofluorescence density for C3 in each group. Scale bar: 50 μm. Student’s unpaired *t*-test. Values are all expressed as mean ± SD. ^**^P < 0.01. ^***^P < 0.001.

Astrocytes could be divided into two different subtypes, termed “A1” and “A2” (Zamanian et al., 2012). Previous studies have demonstrated that A1 astrocytes are induced by activated microglia to produce inflammatory factors to kill neurons. A1 astrocytes have been shown to cluster in the human brain of patients with Alzheimer’s disease, Parkinson’s disease, Huntington’s disease, amyotrophic lateral sclerosis, and multiple sclerosis. A1 astrocytes can be identified by the distribution of AQP4 and expression of C3 (Liddelow et al., 2017). In this study, we demonstrated that administration of MP suppressed the activation of microglia. Hence, we measured the distribution of AQP4 and expression of C3 to determine whether MP could suppress A1 astrocyte activation. Administration of MP suppressed neuronal apoptosis and promoted functional recovery after SCI. As shown in Figure 6, AQP4 protein retained polarization in astrocytes after MP administration compared to SCI mice. Furthermore, the number of C3-positive astrocyte cells was significantly lower in MP-treated mice compared to mice in the SCI group at day 3 post-injury. A previous study showed that Il-1α, TNFα, and C1q together are sufficient to induce the A1 phenotype (Liddelow et al., 2017). To determine whether MP could inhibit the formation of A1 astrocytes, we measured the expression levels of Il-1α and TNFα. We demonstrated that MP treatment could reduce the expression levels of Il-1α and TNFα.

## Discussion

In this study, we demonstrated the mechanism by which MP could have beneficial effects in TSCI mouse models. MP was shown to reduce oxidative damage in astrocyte cells and attenuate neuronal cell death after TSCI *in vivo*. Furthermore, MP could inhibit microglial activation, suppress astrocyte activate to A1sand promote mice TSCI functional recovery.

The glucocorticoid drug MP has been used clinically as an effective therapy for acute TSCI. It alleviates secondary injury by reducing inflammation and ischemic reaction, as well as inhibiting lipid peroxidation (Hall, 2011). The use of MP remains controversial, in part, due to the side effects of infections, bleeding, and femoral head necrosis (Hurlbert and Hamilton, 2008; Miekisiak et al., 2014; Liu et al., 2019). However, the use of MP for the treatment of TSCI has been very effective. MP has been shown to inhibit early inflammatory processes and lipid peroxidation, reduce edema and cell apoptosis, maintain neuronal excitability, and improve microcirculation after TSCI (Rabchevsky et al., 2002; Baltin et al., 2021). The results on the use of MP in animal models have been promising; however, human clinical trials have produced mixed results. MP has been recommended to be administered within 8 h post-injury for the clinical treatment of TSCI (Druschel et al., 2013). Previous studies have demonstrated that MP therapy in TSCI models has a very short therapeutic window. Delayed treatment has shown no effects compared to the saline-treated group (Bracken et al., 1997). MP has been shown to improve neurologic outcomes up to 1-year post-injury if administered within 8 h of injury and in a dosing regimen of bolus 30 mg/kg over 15 min, with maintenance infusion of 5.4 mg/kg per hour infused for 23 h in the clinic (Bracken, 2012). Unfortunately, using this dose also increased the incidence of complications and adverse events (Miekisiak et al., 2014). In the present study, MP was initially administered immediately after TSCI via the tail vein to provide an effective concentration immediately after TSCI. The dose regimen used in this study was bolus 30 mg/kg without maintenance infusion. We observe that the MP group had an increase in GAP-43 expression (axon maker) compared to the SCI group. Fluoro-Jade B staining also showed that the number of degenerated neurons in the MP group was significantly lower compared to the SCI group. These results confirmed that early administration of a single MP pulse therapy (30 mg/kg) immediately after TSCI was effective in promoting functional recovery in our mouse model. However, we did not observe changes in NF-200 expression levels during MP treatment after TSCI. The probable reason may be that NF-200 is an intermediate filament found in the cytoplasm of a neuron. In the adult nervous system, the nerve filaments in small unmyelinated axons contain more peripheral proteins and lower levels of NF-200, while the nerve filaments in large myelinated axons contain more NF-200 and lower levels of peripheral proteins (Molliver et al., 1995; Letournel et al., 2006). In addition to the loss of neurons and astrocytes after TSCI, oligodendrocytes that form the myelin are effectively inhibited, which consequently affects the formation of neuronal myelin. Hence, NF-200 expression levels may not change.

Recent studies have shown that neuroinflammation induced by A1 astrocytes and ischemia promotes the generation of A2 astrocytes (Zamanian et al., 2012). A1 astrocytes are neurotoxic leading to neuronal death, synapse disassembly, and oligodendrocyte death. LPS treatment has been shown to activate microglia to induce the transformation of naïve astrocytes into A1 astrocytes. This is through the secretion of Il-1α, TNF, and C1q cytokines, all of which are essential for inducing A1 astrocytes (Liddelow et al., 2017). In the present study, we demonstrated that administration of MP effectively suppressed the activation of microglia. This was demonstrated by IBA1 immunostaining (represents active microglia). Having demonstrated that A1 astrocytes are activated by microglia, we then investigated whether MP was able to suppress A1 neurotoxic reactive astrocyte activation. A1 reactive astrocytes were observed by immunostaining for C3, an A1 marker. We found that the number of C3-positive astrocytes in the MP group was markedly decreased compared to the TSCI group.

Methylprednisolone is a synthetic anti-inflammatory glucocorticoid classified as a steroidal anti-inflammatory drug. In this study, we demonstrated that MP plays an anti-inflammatory role in TSCI by inhibiting microglial activation, reducing the expression levels of Il-1α, TNFα, and suppressing A1 neurotoxic reactive astrocyte activation in TSCI mouse models. Although C3-positive astrocytes were identified as A1 neurotoxic reactive astrocytes, previous publications have shown that an abnormal expression and distribution of AQP4 protein could indicate the formation of A1 neurotoxic reactive astrocytes (Liddelow et al., 2017). AQP4 is a water channel protein that is highly expressed in peri-micro vessel astrocyte foot processes, glia limits, and ependymal that typically raise the osmotic permeability of the plasma cell membrane (Tait et al., 2008; Saadoun and Papadopoulos, 2010). Overexpression of AQP4 leads to astrocyte swelling and the generation of cytotoxic edema during the early phases of TSCI. Saadoun et al. demonstrated that deletion of AQP4 could reduce spinal cord edema measured 48 h after TSCI and markedly improved neurological outcomes in compression TSCI mouse models (Saadoun et al., 2008). However, AQP4-knockout mice exhibited numerous olfactory and auditory defects, underscoring its importance (Lu et al., 1996, 2008). Recent studies have demonstrated that the polarity distribution of AQP4 could maintain the osmotic permeability of astrocytes (Kitchen et al., 2020). We hypothesize that restoring the polarity distribution of AQP4 protein in astrocytes could promote TSCI repair. Our study demonstrated that the nonpolar distribution of AQP4 protein after TSCI and MP could effectively promote AQP4 protein polarity distribution.

In conclusion, our findings suggest that MP could attenuate astrocyte cell death, decrease microglia activation, and promote functional recovery after acute TSCI in mouse models. We believe that MP promotes the survival of astrocytes to secrete neurotrophic factors and inhibit the surviving astrocytes to transform into the A1 phenotype. MP has several beneficial effects for the treatment of TSCI. However, it is unclear which of these are responsible for its therapeutic effect. Studies have suggested that non-coding RNAs, such as miRNAs and lncRNAs, could inhibit inflammatory diseases (Stagakis et al., 2011; Liu et al., 2020) and MP has been shown to regulate the expression of non-coding RNAs to inhibit specific proinflammatory targets (Davis et al., 2013; Mirzadeh et al., 2019).

## Data Availability Statement

The original contributions presented in the study are included in the article/supplementary material, further inquiries can be directed to the corresponding author.

## Ethics Statement

The animal study was reviewed and approved by Ethic Committee of Animal Research in The Third Affiliated Hospital of Soochow University.

## Author Contributions

All authors contributed substantially to this work. HZ and JL conceived and designed the experiments. S-WG performed the experiments. LZ prepared the figures. HZ wrote the manuscript with the help of LZ. XX helped to purchase the experimental consumables and reagents.

## Conflict of Interest

The authors declare that the research was conducted in the absence of any commercial or financial relationships that could be construed as a potential conflict of interest.
